# 3′,6′-Bis(ethyl­amino)-2′,7′-dimethyl-2-{[2-[(*E*)-3,4-methyl­enedioxy­benzyl­idene­amino]eth­yl}spiro­[isoindoline-1,9′-xanthen]-3-one

**DOI:** 10.1107/S1600536809025872

**Published:** 2009-07-15

**Authors:** Zhi-Hong Xu, Hong-Sheng Wang, Lian-Ting Tao, Hong-Wei Wang

**Affiliations:** a College of Chemistry and Chemical Engineering, Xuchang University, Xuchang, Henan Province 461000, People’s Republic of China; b Institute of Traditional Chinese Materia Medica, Chengde Medical College, Chengde 067000, People’s Republic of China

## Abstract

The title compound, C_36_H_36_N_4_O_4_, was prepared as a spiro­lactam ring formation of the rhodamine dye for comparison with a ring-opened form. The xanthene ring system is approximately planar [r.m.s. deviations from planarity = 0.023 (9) Å for the xanthene ring]. The dihedral angles formed by the spiro­lactam and 1,3-benzodioxole rings with the xanthene ring system are 86.8 (1) and 74.3 (1)°, respectively.

## Related literature

Rhodamine dyes are one of the most widely used fluoro­phores for labeling and sensing biomolecules, see: Ko *et al.* (2006 ▶); Wu *et al.* (2007 ▶). For the structures of rhodamine derivatives bearing a lactam unit, see: Kwon *et al.* (2006 ▶); Wu *et al.* (2007 ▶); Zhang *et al.* (2008 ▶); Deng *et al.* (2009 ▶); Tian & Peng (2008 ▶).
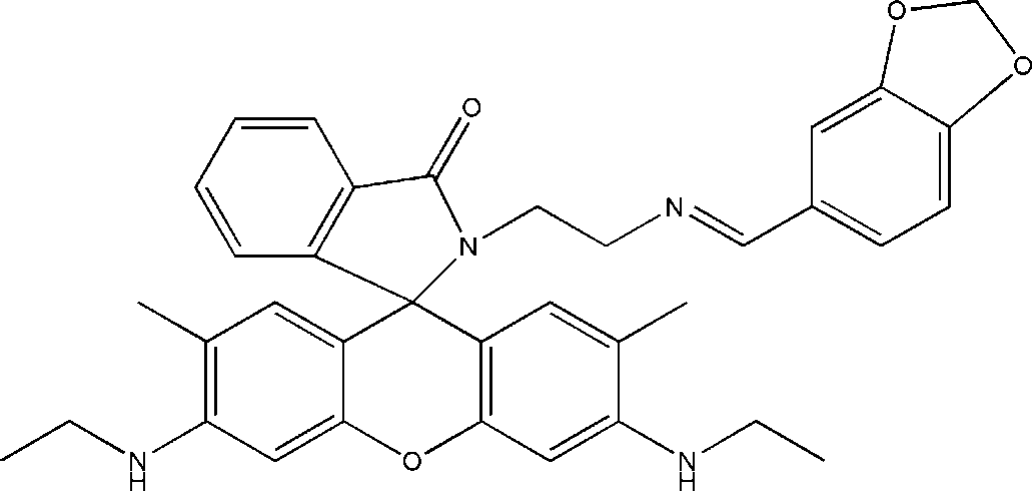


## Experimental

### 

#### Crystal data

C_36_H_36_N_4_O_4_
*M* *_r_* = 588.69
Triclinic, 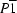

*a* = 9.561 (4) Å
*b* = 12.262 (5) Å
*c* = 13.005 (6) Å
α = 93.623 (8)°
β = 92.078 (8)°
γ = 92.827 (7)°
*V* = 1518.5 (12) Å^3^
*Z* = 2
Mo *K*α radiation
μ = 0.09 mm^−1^
*T* = 296 K
0.35 × 0.32 × 0.27 mm

#### Data collection

Bruker SMART CCD area detector diffractometer
Absorption correction: multi-scan (*SADABS*; Sheldrick, 1996 ▶) *T*_min_ = 0.971, *T*_max_ = 0.977
8102 measured reflections
5606 independent reflections
3838 reflections with *I* > 2σ(*I*)
*R*_int_ = 0.016

#### Refinement

*R*[*F* ^2^ > 2σ(*F* ^2^)] = 0.048
*wR*(*F* ^2^) = 0.069
*S* = 1.83
5606 reflections
409 parameters
H atoms treated by a mixture of independent and constrained refinement
Δρ_max_ = 0.18 e Å^−3^
Δρ_min_ = −0.25 e Å^−3^

### 

Data collection: *SMART* (Bruker, 2005 ▶); cell refinement: *SAINT* (Bruker, 2005 ▶); data reduction: *SAINT*; program(s) used to solve structure: *SHELXS97* (Sheldrick, 2008 ▶); program(s) used to refine structure: *SHELXL97* (Sheldrick, 2008 ▶); molecular graphics: *SHELXTL* (Sheldrick, 2008 ▶); software used to prepare material for publication: *SHELXTL*.

## Supplementary Material

Crystal structure: contains datablocks I, global. DOI: 10.1107/S1600536809025872/at2829sup1.cif

Structure factors: contains datablocks I. DOI: 10.1107/S1600536809025872/at2829Isup2.hkl

Additional supplementary materials:  crystallographic information; 3D view; checkCIF report

## Figures and Tables

**Fig. 1.**
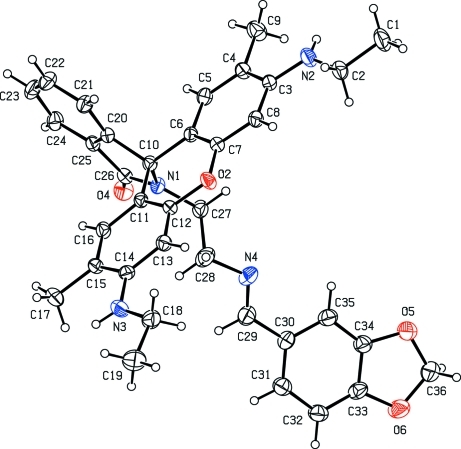
The molecular structure of the title compound, with displacement ellipsoids drawn at 30% probability level.

## supplementary crystallographic information

### Comment

Among many fluorescent compounds, rhodamine dyes are known to have excellent photophysical properties, and they are one of the most widely used fluorophores for labeling and sensing biomolecules (Ko *et al.*, 2006; Wu *et al.*, 2007). There are a few single-crystal reports about rhodamine derivatives bearing a lactam moiety (Kwon *et al.*, 2006; Wu *et al.*, 2007; Zhang *et al.*, 2008; Tian *et al.*, 2008; Deng *et al.*, 2009). Detailed information on their molecular and crystal structures is necessary to understand their photophysical and photochemical properties.

In agreement with other reported models, (Wu *et al.*, 2007; Zhang *et al.*, 2008; Tian *et al.*, 2008;) the main skeleton of the molecule is formed by the xanthene ring and the spirolactam-ring. As shown in Figure 1, The atoms of the xanthene ring or the spirolactam-ring are both nearly planar and are almost perpendicular to each other. The dihedral angle between the xanthene mean planes and the spirolactamring fragment is 86.8°. The dihedral angle between the xanthene mean planes and the 1,3-benzodioxole ring is 74.3°.

### Experimental

A portion of the *N*-(rhodamine-6 *G*)lactam-ethylenediamine (228 mg, 0.5 mmol) and 3,4-methylenedioxy-benzaldehyde (90 mg, 0.6 mmol) were mixed in fresh distilled acetonitrile (50 ml). The reaction solution was refluxed for 24 h under N_2_ atmosphere, the reslulting solution was evaporated to 10 ml and allowed to stand at room temperature overnight. The precipitate which appeared next day was filtered and the crude product was purified by recrystallization from acetonitrile to give 264.6 mg of the title compound in 90% yield. Single crystals suitable for X-ray measurements were obtained from acetonitrile solution by slow evaporation at room temperature.

### Refinement

The H atoms attached to C, N and O atoms were placed in geometrically calculated positions (C—H = 0.93–0.97 Å, N—H = 0.86 Å and O—H = 0.82 Å) and refined as riding, with *U*_iso_(H) = 1.2*U*_eq_(C, N) or 1.5*U*_eq_(methyl C, O).

### Crystal data

**Table:**

C_36_H_36_N_4_O_4_	*Z* = 2
*M**_r_* = 588.69	*F*(000) = 624
Triclinic, *P*1	*D*_x_ = 1.287 Mg m^−^^3^
*a* = 9.561 (4) Å	Mo *K*α radiation, λ = 0.71073 Å
*b* = 12.262 (5) Å	Cell parameters from 2059 reflections
*c* = 13.005 (6) Å	θ = 2.6–25.8°
α = 93.623 (8)°	µ = 0.09 mm^−^^1^
β = 92.078 (8)°	*T* = 296 K
γ = 92.827 (7)°	Block, colorless
*V* = 1518.5 (12) Å^3^	0.35 × 0.32 × 0.27 mm

### Data collection

**Table:**

Bruker SMART CCD area detector diffractometer	5606 independent reflections
Radiation source: fine-focus sealed tube	3838 reflections with *I* > 2σ(*I*)
graphite	*R*_int_ = 0.016
φ and ω scans	θ_max_ = 25.5°, θ_min_ = 1.6°
Absorption correction: multi-scan (*SADABS*; Sheldrick, 1996)	*h* = −11→11
*T*_min_ = 0.971, *T*_max_ = 0.977	*k* = −13→14
8102 measured reflections	*l* = −13→15

### Refinement

**Table:**

Refinement on *F*^2^	Primary atom site location: structure-invariant direct methods
Least-squares matrix: full	Secondary atom site location: difference Fourier map
*R*[*F*^2^ > 2σ(*F*^2^)] = 0.048	Hydrogen site location: inferred from neighbouring sites
*wR*(*F*^2^) = 0.069	H atoms treated by a mixture of independent and constrained refinement
*S* = 1.83	*w* = 1/[σ^2^(*F*_o_^2^)]
5606 reflections	(Δ/σ)_max_ < 0.001
409 parameters	Δρ_max_ = 0.18 e Å^−^^3^
0 restraints	Δρ_min_ = −0.25 e Å^−^^3^

### Special details

**Table:**

Geometry. All e.s.d.'s (except the e.s.d. in the dihedral angle between two l.s. planes) are estimated using the full covariance matrix. The cell e.s.d.'s are taken into account individually in the estimation of e.s.d.'s in distances, angles and torsion angles; correlations between e.s.d.'s in cell parameters are only used when they are defined by crystal symmetry. An approximate (isotropic) treatment of cell e.s.d.'s is used for estimating e.s.d.'s involving l.s. planes.
Refinement. Refinement of *F*^2^ against ALL reflections. The weighted *R*-factor *wR* and goodness of fit *S* are based on *F*^2^, conventional *R*-factors *R* are based on *F*, with *F* set to zero for negative *F*^2^. The threshold expression of *F*^2^ > σ(*F*^2^) is used only for calculating *R*-factors(gt) *etc*. and is not relevant to the choice of reflections for refinement. *R*-factors based on *F*^2^ are statistically about twice as large as those based on *F*, and *R*- factors based on ALL data will be even larger.

### Fractional atomic coordinates and isotropic or equivalent isotropic displacement parameters (Å^2^)

**Table:**

	*x*	*y*	*z*	*U*_iso_*/*U*_eq_	
C1	1.0868 (2)	1.00378 (15)	−0.21394 (16)	0.0673 (7)	
H1A	1.0771	1.0680	−0.1692	0.101*	
H1B	1.0286	1.0075	−0.2752	0.101*	
H1C	1.1828	0.9996	−0.2323	0.101*	
C2	1.0422 (2)	0.90267 (15)	−0.15876 (15)	0.0498 (6)	
H2A	0.9451	0.9067	−0.1404	0.060*	
H2B	1.0499	0.8378	−0.2044	0.060*	
C3	1.1025 (2)	0.81736 (15)	0.00388 (15)	0.0366 (5)	
C4	1.1834 (2)	0.82230 (15)	0.09733 (15)	0.0384 (5)	
C5	1.15344 (19)	0.74560 (14)	0.16730 (14)	0.0384 (5)	
H5	1.2072	0.7484	0.2285	0.046*	
C6	1.04600 (19)	0.66337 (14)	0.15110 (14)	0.0317 (5)	
C7	0.97058 (19)	0.66070 (14)	0.05864 (14)	0.0333 (5)	
C8	0.99800 (19)	0.73535 (14)	−0.01517 (14)	0.0373 (5)	
H8	0.9462	0.7303	−0.0774	0.045*	
C9	1.2989 (2)	0.91013 (15)	0.11943 (16)	0.0585 (6)	
H9A	1.2585	0.9796	0.1321	0.088*	
H9B	1.3576	0.9130	0.0612	0.088*	
H9C	1.3540	0.8936	0.1791	0.088*	
C10	1.01167 (19)	0.58503 (14)	0.23360 (14)	0.0329 (5)	
C11	0.89724 (18)	0.50054 (14)	0.19301 (14)	0.0312 (5)	
C12	0.82788 (19)	0.50651 (14)	0.09922 (15)	0.0335 (5)	
C13	0.71638 (19)	0.43470 (14)	0.06531 (15)	0.0382 (5)	
H13	0.6713	0.4423	0.0018	0.046*	
C14	0.6722 (2)	0.35141 (15)	0.12629 (15)	0.0371 (5)	
C15	0.7419 (2)	0.34181 (15)	0.22286 (15)	0.0381 (5)	
C16	0.85157 (19)	0.41539 (14)	0.25314 (14)	0.0381 (5)	
H16	0.8976	0.4083	0.3164	0.046*	
C17	0.6979 (2)	0.25228 (15)	0.29171 (16)	0.0565 (6)	
H17A	0.7611	0.2543	0.3509	0.085*	
H17B	0.6999	0.1824	0.2542	0.085*	
H17C	0.6046	0.2633	0.3137	0.085*	
C18	0.4790 (2)	0.28637 (16)	−0.00008 (17)	0.0574 (6)	
H18A	0.5399	0.2817	−0.0581	0.069*	
H18B	0.4365	0.3566	0.0010	0.069*	
C19	0.3653 (2)	0.19525 (17)	−0.01317 (19)	0.0815 (8)	
H19A	0.4075	0.1257	−0.0159	0.122*	
H19B	0.3116	0.2023	−0.0760	0.122*	
H19C	0.3049	0.2001	0.0441	0.122*	
C20	1.14037 (19)	0.53312 (14)	0.27722 (14)	0.0320 (5)	
C21	1.2363 (2)	0.47156 (14)	0.22513 (15)	0.0426 (5)	
H21	1.2253	0.4545	0.1545	0.051*	
C22	1.3490 (2)	0.43615 (15)	0.28146 (17)	0.0507 (6)	
H22	1.4161	0.3964	0.2478	0.061*	
C23	1.3639 (2)	0.45904 (16)	0.38774 (17)	0.0503 (6)	
H23	1.4399	0.4338	0.4243	0.060*	
C24	1.2665 (2)	0.51889 (15)	0.43903 (15)	0.0445 (5)	
H24	1.2754	0.5340	0.5100	0.053*	
C25	1.15530 (19)	0.55590 (14)	0.38242 (14)	0.0332 (5)	
C26	1.0429 (2)	0.62796 (15)	0.41636 (16)	0.0363 (5)	
C27	0.86305 (19)	0.72948 (14)	0.32757 (15)	0.0435 (5)	
H27A	0.8826	0.7745	0.2706	0.052*	
H27B	0.8758	0.7761	0.3906	0.052*	
C28	0.7120 (2)	0.68842 (16)	0.31699 (18)	0.0617 (7)	
H28A	0.6937	0.6478	0.2510	0.074*	
H28B	0.6904	0.6404	0.3714	0.074*	
C29	0.5234 (2)	0.77978 (17)	0.38177 (17)	0.0555 (6)	
H29	0.5086	0.7161	0.4160	0.067*	
C30	0.4246 (2)	0.86726 (16)	0.39934 (16)	0.0446 (5)	
C31	0.3017 (2)	0.84294 (16)	0.44650 (16)	0.0523 (6)	
H31	0.2865	0.7731	0.4692	0.063*	
C32	0.1989 (2)	0.91831 (17)	0.46180 (17)	0.0558 (6)	
H32	0.1159	0.9008	0.4936	0.067*	
C33	0.2274 (2)	1.01850 (17)	0.42755 (17)	0.0503 (6)	
C34	0.3508 (2)	1.04583 (17)	0.38140 (17)	0.0491 (6)	
C35	0.4526 (2)	0.97297 (16)	0.36598 (16)	0.0510 (6)	
H35	0.5359	0.9920	0.3352	0.061*	
C36	0.2187 (2)	1.19285 (17)	0.38506 (19)	0.0656 (7)	
H36A	0.2343	1.2557	0.4340	0.079*	
H36B	0.1657	1.2152	0.3256	0.079*	
N1	0.96564 (15)	0.64561 (11)	0.32967 (11)	0.0345 (4)	
N4	0.62548 (19)	0.78474 (14)	0.32444 (15)	0.0602 (5)	
N2	1.1293 (2)	0.89507 (15)	−0.06710 (14)	0.0502 (5)	
O2	0.86117 (13)	0.58540 (10)	0.03119 (9)	0.0419 (4)	
N3	0.56092 (19)	0.27858 (15)	0.09466 (16)	0.0523 (5)	
O4	1.02493 (14)	0.66716 (10)	0.50372 (10)	0.0505 (4)	
O5	0.34971 (16)	1.15342 (11)	0.35415 (14)	0.0777 (5)	
O6	0.14296 (15)	1.10722 (12)	0.43125 (13)	0.0706 (5)	
H3N	0.542 (2)	0.2270 (15)	0.1319 (15)	0.061 (8)*	
H2N	1.183 (2)	0.9440 (15)	−0.0491 (15)	0.057 (8)*	

### Atomic displacement parameters (Å^2^)

**Table:**

	*U*^11^	*U*^22^	*U*^33^	*U*^12^	*U*^13^	*U*^23^
C1	0.0826 (19)	0.0578 (15)	0.0642 (17)	−0.0007 (13)	−0.0017 (14)	0.0310 (13)
C2	0.0529 (15)	0.0500 (13)	0.0481 (14)	0.0009 (11)	−0.0011 (12)	0.0188 (12)
C3	0.0382 (12)	0.0331 (11)	0.0402 (13)	0.0051 (10)	0.0070 (10)	0.0088 (10)
C4	0.0409 (13)	0.0360 (12)	0.0385 (13)	−0.0013 (10)	0.0005 (10)	0.0051 (10)
C5	0.0401 (13)	0.0424 (12)	0.0325 (12)	0.0044 (10)	−0.0059 (10)	0.0037 (10)
C6	0.0332 (11)	0.0316 (11)	0.0309 (12)	0.0044 (9)	0.0010 (9)	0.0049 (9)
C7	0.0339 (12)	0.0315 (11)	0.0350 (12)	0.0006 (10)	0.0021 (10)	0.0059 (10)
C8	0.0384 (12)	0.0409 (12)	0.0331 (12)	0.0024 (10)	−0.0029 (9)	0.0082 (10)
C9	0.0613 (16)	0.0547 (14)	0.0579 (15)	−0.0132 (12)	−0.0068 (12)	0.0092 (12)
C10	0.0357 (12)	0.0355 (11)	0.0283 (11)	0.0048 (10)	0.0024 (9)	0.0050 (9)
C11	0.0341 (12)	0.0310 (11)	0.0292 (11)	0.0049 (9)	0.0038 (9)	0.0037 (9)
C12	0.0328 (12)	0.0328 (11)	0.0359 (12)	0.0016 (9)	0.0051 (10)	0.0081 (10)
C13	0.0371 (12)	0.0413 (12)	0.0364 (12)	0.0001 (10)	−0.0019 (10)	0.0072 (10)
C14	0.0320 (12)	0.0347 (12)	0.0451 (13)	0.0025 (10)	0.0063 (10)	0.0022 (11)
C15	0.0386 (12)	0.0358 (12)	0.0416 (13)	0.0032 (10)	0.0099 (10)	0.0087 (11)
C16	0.0428 (13)	0.0399 (12)	0.0332 (12)	0.0069 (10)	0.0030 (10)	0.0094 (10)
C17	0.0577 (15)	0.0544 (14)	0.0592 (16)	−0.0044 (12)	0.0067 (12)	0.0218 (13)
C18	0.0439 (14)	0.0501 (14)	0.0772 (18)	−0.0020 (12)	−0.0104 (13)	0.0071 (13)
C19	0.0530 (16)	0.0713 (17)	0.116 (2)	−0.0197 (14)	−0.0203 (15)	0.0078 (16)
C20	0.0331 (11)	0.0314 (11)	0.0320 (12)	0.0027 (9)	0.0007 (9)	0.0059 (9)
C21	0.0434 (13)	0.0459 (13)	0.0393 (13)	0.0090 (11)	0.0005 (11)	0.0042 (11)
C22	0.0435 (14)	0.0530 (14)	0.0571 (16)	0.0172 (11)	0.0045 (12)	0.0016 (12)
C23	0.0416 (13)	0.0510 (14)	0.0588 (16)	0.0122 (11)	−0.0117 (12)	0.0073 (12)
C24	0.0492 (14)	0.0470 (13)	0.0370 (13)	0.0040 (11)	−0.0079 (11)	0.0058 (11)
C25	0.0361 (12)	0.0316 (11)	0.0321 (12)	0.0000 (9)	−0.0002 (10)	0.0066 (9)
C26	0.0401 (13)	0.0342 (12)	0.0346 (13)	−0.0024 (10)	0.0028 (10)	0.0058 (10)
C27	0.0482 (14)	0.0399 (12)	0.0443 (13)	0.0117 (11)	0.0080 (11)	0.0056 (10)
C28	0.0477 (15)	0.0549 (15)	0.0834 (19)	0.0167 (12)	0.0039 (13)	−0.0004 (13)
C29	0.0504 (15)	0.0536 (14)	0.0631 (17)	0.0079 (12)	−0.0034 (13)	0.0074 (13)
C30	0.0440 (14)	0.0455 (13)	0.0439 (14)	0.0069 (11)	−0.0032 (11)	−0.0007 (11)
C31	0.0527 (15)	0.0509 (14)	0.0539 (15)	0.0005 (12)	0.0080 (12)	0.0064 (12)
C32	0.0485 (15)	0.0524 (15)	0.0676 (17)	0.0009 (12)	0.0174 (13)	0.0056 (13)
C33	0.0404 (14)	0.0482 (14)	0.0617 (16)	0.0082 (12)	0.0063 (12)	−0.0073 (12)
C34	0.0462 (15)	0.0432 (14)	0.0579 (16)	0.0008 (12)	0.0061 (12)	0.0008 (12)
C35	0.0411 (14)	0.0562 (15)	0.0562 (15)	0.0040 (12)	0.0075 (11)	0.0027 (12)
C36	0.0593 (17)	0.0528 (15)	0.087 (2)	0.0119 (13)	0.0100 (14)	0.0092 (14)
N1	0.0379 (10)	0.0356 (9)	0.0309 (10)	0.0095 (8)	0.0021 (8)	0.0032 (8)
N4	0.0460 (12)	0.0657 (13)	0.0716 (15)	0.0197 (10)	0.0092 (11)	0.0086 (11)
N2	0.0550 (13)	0.0457 (13)	0.0504 (13)	−0.0078 (11)	−0.0064 (10)	0.0197 (11)
O2	0.0443 (9)	0.0436 (8)	0.0374 (8)	−0.0103 (7)	−0.0093 (7)	0.0154 (7)
N3	0.0471 (12)	0.0488 (13)	0.0613 (14)	−0.0095 (10)	0.0016 (10)	0.0159 (12)
O4	0.0631 (10)	0.0582 (9)	0.0303 (8)	0.0083 (8)	0.0046 (7)	−0.0029 (7)
O5	0.0602 (11)	0.0485 (10)	0.1288 (16)	0.0102 (9)	0.0299 (11)	0.0207 (10)
O6	0.0518 (10)	0.0517 (10)	0.1091 (15)	0.0076 (8)	0.0208 (10)	−0.0020 (10)

### Geometric parameters (Å, °)

**Table:**

C1—C2	1.522 (2)	C19—H19A	0.9600
C1—H1A	0.9600	C19—H19B	0.9600
C1—H1B	0.9600	C19—H19C	0.9600
C1—H1C	0.9600	C20—C25	1.380 (2)
C2—N2	1.440 (2)	C20—C21	1.385 (2)
C2—H2A	0.9700	C21—C22	1.384 (3)
C2—H2B	0.9700	C21—H21	0.9300
C3—C8	1.386 (2)	C22—C23	1.394 (3)
C3—N2	1.389 (2)	C22—H22	0.9300
C3—C4	1.413 (3)	C23—C24	1.379 (3)
C4—C5	1.377 (2)	C23—H23	0.9300
C4—C9	1.511 (2)	C24—C25	1.382 (2)
C5—C6	1.404 (2)	C24—H24	0.9300
C5—H5	0.9300	C25—C26	1.486 (2)
C6—C7	1.377 (2)	C26—O4	1.227 (2)
C6—C10	1.519 (2)	C26—N1	1.360 (2)
C7—O2	1.3821 (19)	C27—N1	1.456 (2)
C7—C8	1.391 (2)	C27—C28	1.504 (2)
C8—H8	0.9300	C27—H27A	0.9700
C9—H9A	0.9600	C27—H27B	0.9700
C9—H9B	0.9600	C28—N4	1.475 (2)
C9—H9C	0.9600	C28—H28A	0.9700
C10—N1	1.504 (2)	C28—H28B	0.9700
C10—C20	1.521 (2)	C29—N4	1.251 (2)
C10—C11	1.525 (2)	C29—C30	1.476 (3)
C11—C12	1.375 (2)	C29—H29	0.9300
C11—C16	1.405 (2)	C30—C31	1.373 (2)
C12—O2	1.3850 (19)	C30—C35	1.409 (2)
C12—C13	1.389 (2)	C31—C32	1.393 (3)
C13—C14	1.391 (2)	C31—H31	0.9300
C13—H13	0.9300	C32—C33	1.351 (2)
C14—N3	1.388 (2)	C32—H32	0.9300
C14—C15	1.415 (3)	C33—C34	1.380 (2)
C15—C16	1.378 (2)	C33—O6	1.386 (2)
C15—C17	1.513 (2)	C34—C35	1.365 (3)
C16—H16	0.9300	C34—O5	1.388 (2)
C17—H17A	0.9600	C35—H35	0.9300
C17—H17B	0.9600	C36—O6	1.423 (2)
C17—H17C	0.9600	C36—O5	1.427 (2)
C18—N3	1.447 (3)	C36—H36A	0.9700
C18—C19	1.517 (2)	C36—H36B	0.9700
C18—H18A	0.9700	N2—H2N	0.790 (17)
C18—H18B	0.9700	N3—H3N	0.836 (17)
			
C2—C1—H1A	109.5	H19A—C19—H19C	109.5
C2—C1—H1B	109.5	H19B—C19—H19C	109.5
H1A—C1—H1B	109.5	C25—C20—C21	120.81 (18)
C2—C1—H1C	109.5	C25—C20—C10	110.72 (16)
H1A—C1—H1C	109.5	C21—C20—C10	128.47 (18)
H1B—C1—H1C	109.5	C22—C21—C20	117.94 (19)
N2—C2—C1	110.27 (17)	C22—C21—H21	121.0
N2—C2—H2A	109.6	C20—C21—H21	121.0
C1—C2—H2A	109.6	C21—C22—C23	121.21 (19)
N2—C2—H2B	109.6	C21—C22—H22	119.4
C1—C2—H2B	109.6	C23—C22—H22	119.4
H2A—C2—H2B	108.1	C24—C23—C22	120.3 (2)
C8—C3—N2	121.31 (19)	C24—C23—H23	119.8
C8—C3—C4	119.48 (17)	C22—C23—H23	119.8
N2—C3—C4	119.21 (18)	C23—C24—C25	118.43 (19)
C5—C4—C3	118.24 (18)	C23—C24—H24	120.8
C5—C4—C9	121.26 (18)	C25—C24—H24	120.8
C3—C4—C9	120.50 (17)	C20—C25—C24	121.28 (18)
C4—C5—C6	123.46 (18)	C20—C25—C26	109.15 (17)
C4—C5—H5	118.3	C24—C25—C26	129.45 (19)
C6—C5—H5	118.3	O4—C26—N1	126.31 (19)
C7—C6—C5	116.48 (16)	O4—C26—C25	127.68 (19)
C7—C6—C10	122.18 (17)	N1—C26—C25	105.97 (17)
C5—C6—C10	121.29 (17)	N1—C27—C28	115.78 (15)
C6—C7—O2	123.60 (16)	N1—C27—H27A	108.3
C6—C7—C8	122.26 (18)	C28—C27—H27A	108.3
O2—C7—C8	114.13 (17)	N1—C27—H27B	108.3
C3—C8—C7	120.05 (18)	C28—C27—H27B	108.3
C3—C8—H8	120.0	H27A—C27—H27B	107.4
C7—C8—H8	120.0	N4—C28—C27	107.44 (16)
C4—C9—H9A	109.5	N4—C28—H28A	110.2
C4—C9—H9B	109.5	C27—C28—H28A	110.2
H9A—C9—H9B	109.5	N4—C28—H28B	110.2
C4—C9—H9C	109.5	C27—C28—H28B	110.2
H9A—C9—H9C	109.5	H28A—C28—H28B	108.5
H9B—C9—H9C	109.5	N4—C29—C30	125.0 (2)
N1—C10—C6	111.05 (14)	N4—C29—H29	117.5
N1—C10—C20	99.76 (14)	C30—C29—H29	117.5
C6—C10—C20	112.95 (15)	C31—C30—C35	120.1 (2)
N1—C10—C11	109.94 (14)	C31—C30—C29	118.63 (19)
C6—C10—C11	110.04 (15)	C35—C30—C29	121.19 (19)
C20—C10—C11	112.71 (14)	C30—C31—C32	122.9 (2)
C12—C11—C16	116.36 (17)	C30—C31—H31	118.6
C12—C11—C10	122.45 (16)	C32—C31—H31	118.6
C16—C11—C10	121.10 (17)	C33—C32—C31	115.79 (19)
C11—C12—O2	123.13 (17)	C33—C32—H32	122.1
C11—C12—C13	122.70 (17)	C31—C32—H32	122.1
O2—C12—C13	114.16 (17)	C32—C33—C34	122.6 (2)
C12—C13—C14	120.03 (19)	C32—C33—O6	127.6 (2)
C12—C13—H13	120.0	C34—C33—O6	109.75 (19)
C14—C13—H13	120.0	C35—C34—C33	122.3 (2)
N3—C14—C13	121.38 (19)	C35—C34—O5	128.24 (19)
N3—C14—C15	119.65 (18)	C33—C34—O5	109.46 (19)
C13—C14—C15	118.97 (18)	C34—C35—C30	116.26 (19)
C16—C15—C14	118.74 (17)	C34—C35—H35	121.9
C16—C15—C17	120.40 (19)	C30—C35—H35	121.9
C14—C15—C17	120.86 (18)	O6—C36—O5	108.36 (16)
C15—C16—C11	123.19 (18)	O6—C36—H36A	110.0
C15—C16—H16	118.4	O5—C36—H36A	110.0
C11—C16—H16	118.4	O6—C36—H36B	110.0
C15—C17—H17A	109.5	O5—C36—H36B	110.0
C15—C17—H17B	109.5	H36A—C36—H36B	108.4
H17A—C17—H17B	109.5	C26—N1—C27	122.31 (16)
C15—C17—H17C	109.5	C26—N1—C10	114.23 (15)
H17A—C17—H17C	109.5	C27—N1—C10	122.72 (15)
H17B—C17—H17C	109.5	C29—N4—C28	116.83 (19)
N3—C18—C19	110.73 (17)	C3—N2—C2	122.46 (19)
N3—C18—H18A	109.5	C3—N2—H2N	116.6 (15)
C19—C18—H18A	109.5	C2—N2—H2N	119.4 (15)
N3—C18—H18B	109.5	C7—O2—C12	118.22 (14)
C19—C18—H18B	109.5	C14—N3—C18	123.18 (18)
H18A—C18—H18B	108.1	C14—N3—H3N	117.7 (15)
C18—C19—H19A	109.5	C18—N3—H3N	119.1 (15)
C18—C19—H19B	109.5	C34—O5—C36	106.19 (15)
H19A—C19—H19B	109.5	C33—O6—C36	106.24 (16)
C18—C19—H19C	109.5		
			
C8—C3—C4—C5	−1.0 (3)	C10—C20—C25—C24	−178.84 (15)
N2—C3—C4—C5	178.93 (17)	C21—C20—C25—C26	176.84 (15)
C8—C3—C4—C9	179.32 (17)	C10—C20—C25—C26	−2.4 (2)
N2—C3—C4—C9	−0.7 (3)	C23—C24—C25—C20	0.6 (3)
C3—C4—C5—C6	−0.6 (3)	C23—C24—C25—C26	−174.98 (17)
C9—C4—C5—C6	179.08 (17)	C20—C25—C26—O4	−178.01 (18)
C4—C5—C6—C7	1.2 (3)	C24—C25—C26—O4	−2.0 (3)
C4—C5—C6—C10	−176.25 (17)	C20—C25—C26—N1	−0.2 (2)
C5—C6—C7—O2	−179.61 (15)	C24—C25—C26—N1	175.78 (18)
C10—C6—C7—O2	−2.2 (3)	N1—C27—C28—N4	175.74 (17)
C5—C6—C7—C8	−0.3 (3)	N4—C29—C30—C31	166.7 (2)
C10—C6—C7—C8	177.18 (16)	N4—C29—C30—C35	−11.4 (4)
N2—C3—C8—C7	−178.01 (17)	C35—C30—C31—C32	1.4 (3)
C4—C3—C8—C7	1.9 (3)	C29—C30—C31—C32	−176.7 (2)
C6—C7—C8—C3	−1.3 (3)	C30—C31—C32—C33	−0.3 (3)
O2—C7—C8—C3	178.12 (15)	C31—C32—C33—C34	−0.8 (3)
C7—C6—C10—N1	−115.91 (19)	C31—C32—C33—O6	178.7 (2)
C5—C6—C10—N1	61.4 (2)	C32—C33—C34—C35	0.8 (4)
C7—C6—C10—C20	132.98 (18)	O6—C33—C34—C35	−178.8 (2)
C5—C6—C10—C20	−49.7 (2)	C32—C33—C34—O5	179.7 (2)
C7—C6—C10—C11	6.1 (2)	O6—C33—C34—O5	0.1 (3)
C5—C6—C10—C11	−176.62 (16)	C33—C34—C35—C30	0.4 (3)
N1—C10—C11—C12	115.71 (19)	O5—C34—C35—C30	−178.4 (2)
C6—C10—C11—C12	−6.9 (2)	C31—C30—C35—C34	−1.4 (3)
C20—C10—C11—C12	−133.97 (18)	C29—C30—C35—C34	176.7 (2)
N1—C10—C11—C16	−60.5 (2)	O4—C26—N1—C27	10.4 (3)
C6—C10—C11—C16	176.87 (15)	C25—C26—N1—C27	−167.46 (14)
C20—C10—C11—C16	49.8 (2)	O4—C26—N1—C10	−179.26 (17)
C16—C11—C12—O2	−179.68 (15)	C25—C26—N1—C10	2.92 (19)
C10—C11—C12—O2	3.9 (3)	C28—C27—N1—C26	−110.2 (2)
C16—C11—C12—C13	1.5 (3)	C28—C27—N1—C10	80.2 (2)
C10—C11—C12—C13	−174.94 (16)	C6—C10—N1—C26	−123.48 (17)
C11—C12—C13—C14	−1.1 (3)	C20—C10—N1—C26	−4.13 (18)
O2—C12—C13—C14	179.95 (15)	C11—C10—N1—C26	114.50 (17)
C12—C13—C14—N3	179.83 (17)	C6—C10—N1—C27	46.8 (2)
C12—C13—C14—C15	0.4 (3)	C20—C10—N1—C27	166.20 (14)
N3—C14—C15—C16	−179.57 (17)	C11—C10—N1—C27	−75.17 (19)
C13—C14—C15—C16	−0.1 (3)	C30—C29—N4—C28	−179.9 (2)
N3—C14—C15—C17	0.7 (3)	C27—C28—N4—C29	−133.9 (2)
C13—C14—C15—C17	−179.81 (16)	C8—C3—N2—C2	6.7 (3)
C14—C15—C16—C11	0.5 (3)	C4—C3—N2—C2	−173.21 (19)
C17—C15—C16—C11	−179.76 (16)	C1—C2—N2—C3	172.91 (18)
C12—C11—C16—C15	−1.2 (3)	C6—C7—O2—C12	−1.7 (2)
C10—C11—C16—C15	175.26 (17)	C8—C7—O2—C12	178.86 (15)
N1—C10—C20—C25	3.82 (18)	C11—C12—O2—C7	0.9 (2)
C6—C10—C20—C25	121.76 (17)	C13—C12—O2—C7	179.80 (15)
C11—C10—C20—C25	−112.75 (17)	C13—C14—N3—C18	−3.1 (3)
N1—C10—C20—C21	−175.41 (17)	C15—C14—N3—C18	176.40 (19)
C6—C10—C20—C21	−57.5 (2)	C19—C18—N3—C14	179.95 (18)
C11—C10—C20—C21	68.0 (2)	C35—C34—O5—C36	178.7 (2)
C25—C20—C21—C22	−1.6 (3)	C33—C34—O5—C36	−0.2 (3)
C10—C20—C21—C22	177.57 (17)	O6—C36—O5—C34	0.2 (3)
C20—C21—C22—C23	1.7 (3)	C32—C33—O6—C36	−179.5 (2)
C21—C22—C23—C24	−0.7 (3)	C34—C33—O6—C36	0.0 (3)
C22—C23—C24—C25	−0.5 (3)	O5—C36—O6—C33	−0.2 (2)
C21—C20—C25—C24	0.5 (3)		
